# The mitophagy-inflammasome axis: a shared pathological hub in Alzheimer’s and Parkinson’s diseases

**DOI:** 10.1186/s40035-026-00578-w

**Published:** 2026-08-17

**Authors:** Wei Long, Mengqin Yuan, Sirui Wang, Xinyue Tan, Li-Chen Gao

**Affiliations:** 1 Department of Pharmacy, Phase I Clinical Trial Centre, School of Pharmaceutical Science, Hengyang Medical School, The Affiliated Changsha Central Hospital, University of South China, Changsha, 410004 China; 2 Hunan Provincial Key Laboratory of Tumor Microenvironment Responsive Drug Research, Hengyang, 421001 China; 3 School of Life Science, Hunan University of Science and Technology, Xiangtan, 411201 China

**Keywords:** Mitophagy, Inflammasome, β-Amyloid, α-Synuclein, Targeted therapy

## Abstract

Alzheimer’s disease (AD) and Parkinson’s disease (PD) represent the most prevalent chronic neurodegenerative disorders, characterized by progressive loss of neurons as a core pathological feature. Despite discrepancies in their clinical phenotypes and signature pathological proteins, accumulating evidence has validated a common molecular pathogenic mechanism: dysfunctional bidirectional crosstalk between mitophagy and inflammasomes. As the central hub of neuronal energy metabolism, mitochondrial impairment triggers the release of damage-associated molecular patterns such as reactive oxygen species and mitochondrial DNA, which in turn activate inflammasomes (e.g., NLRP3) to elicit chronic neuroinflammation. Conversely, excessive inflammasome activation suppresses mitophagy, exacerbating the accumulation of damaged mitochondria and pathological protein aggregates, and forming a pathological mitochondrial damage—inflammatory activation—autophagy inhibition cycle. Microglia and astrocytes, key immunocompetent cells of the central nervous system, act as a hub within this regulatory network. Therapeutic strategies targeting the mitophagy-inflammasome axis have achieved remarkable advancements, including mitophagy agonists, inflammasome inhibitors, and dual-target modulators. This review summarizes recent findings regarding the pathogenic roles of β-amyloid and α-synuclein in AD and PD, as well as the protective effects offered by regulating mitophagy and inflammasome activity. Furthermore, the major directions and potential hurdles in the development of targeted therapeutics are discussed, in the aim of providing insights into the novel therapeutic avenues for the treatment of both disorders.

**Graphical abstract:**

**Figure:**
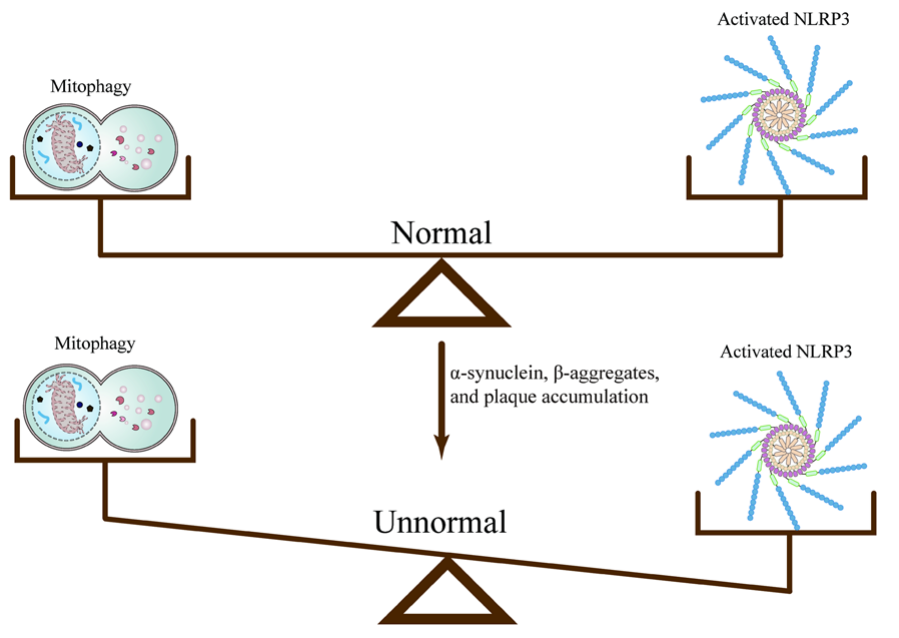

## Introduction

Alzheimer’s disease (AD) and Parkinson’s disease (PD) are prevalent chronic neurodegenerative disorders that pose significant health challenges to the aging global population. They are characterized by progressive neuronal degeneration in the central nervous system (CNS), leading to substantial deficits in cognitive, motor, and other physiological functions [1, 2]. Due to the aging of the global population, the incidence of chronic diseases is rising, imposing significant burdens on socioeconomic systems and human health [3, 4]. Epidemiological analysis indicates that the number of individuals with AD will reach approximately 130 million by 2050 [5]. Furthermore, demographic aging-driven projection models estimate that the global number of individuals with PD will reach 17–17.5 million by 2040, with a median estimate of approximately 17.2 million, representing a doubling of cases within just two decades [6]. As symptoms typically manifest only after neurodegenerative changes have become irreversible, conventional treatments often yield suboptimal outcomes [7]. Pathologically, AD is characterized by senile plaques formed by extracellular β-amyloid (Aβ) deposition and neurofibrillary tangles composed of intracellular hyperphosphorylated tau protein [8]. PD is characterized by progressive degeneration of dopaminergic neurons in the substantia nigra pars compacta, accompanied by intracellular Lewy bodies and Lewy neurites from abnormal α-synuclein (α-Syn) aggregation [9]. Clinically, the cardinal clinical manifestations of AD include progressive memory loss and global cognitive decline, while PD is clinically characterized by core motor symptoms including resting tremor, bradykinesia and muscular rigidity, and non-motor symptoms such as hyposmia and constipation that often precede the onset of motor symptoms [10]. However, a growing body of evidence shows striking commonalities in molecular pathogenesis, namely the mutual promotion of mitochondrial dysfunction and chronic neuroinflammation [11, 12].

Mitochondria serve as the core organelles for energy production, calcium buffering, and redox homeostasis, and are therefore essential for neuronal survival [13]. The high energy demand of neurons renders them particularly vulnerable to mitochondrial injury. When mitochondrial oxidative phosphorylation (OXPHOS) efficiency declines, ATP production becomes insufficient and excessive reactive oxygen species (ROS) and mitochondrial DNA (mtDNA) are released as damage-associated molecular patterns (DAMPs), triggering oxidative stress and inflammatory cascades [14, 15]. Mitophagy, a selective autophagic process that removes damaged mitochondria, is fundamental for maintaining cellular and neuronal homeostasis. The PTEN-induced kinase 1 (PINK1)/Parkin-dependent pathway, one of the best-characterized mechanisms of mitophagy, plays a pivotal role in preserving mitochondrial integrity and neuronal function [16].

Inflammasomes are multi-protein complexes that mediate innate immune signaling and act as central regulators of neuroinflammation [17]. The NOD-, LRR-, and pyrin domain-containing protein 3 (NLRP3) inflammasome is the most extensively studied inflammasome. Aberrant NLRP3 activation leads to caspase-1-mediated maturation of pro-inflammatory cytokines interleukin (IL)-1β and IL-18 and induces pyroptosis, a form of inflammatory cell death. Recent studies have highlighted a bidirectional regulatory relationship between mitophagy and inflammasome activation: defective mitophagy results in the accumulation of mtDNA and ROS that activate NLRP3, whereas excessive inflammasome activation suppresses autophagic flux, further disrupting the mitochondrial quality control [18–20], accelerating neuronal degeneration.

In both AD and PD, pathological proteins such as Aβ and α-Syn not only induce mitochondrial damage, but also activate inflammasomes, amplifying neuroinflammation and neuronal death [21]. Microglia and astrocytes, key immune effector cells in the CNS, serve as critical hubs of this regulatory network by releasing cytokines, chemokines, and complement factors while being modulated by mitochondrial metabolic status [22]. Recent multi-omics studies further revealed a significant overlap between the molecular networks of AD and PD, suggesting that targeting the mitophagy–inflammasome axis may provide a shared therapeutic strategy for these disorders [23].

In this review, we summarize current advances in understanding the bidirectional regulation between mitophagy and inflammasomes, particularly in AD and PD, and discuss translational strategies targeting this regulatory network. A deeper understanding of the dynamic balance within the mitophagy–inflammasome axis will offer new insights into the common pathological basis of neurodegenerative diseases and open new avenues for the development of disease-modifying therapies.

## Mitochondrial dysfunction and mitophagy

**Fig. 1:**
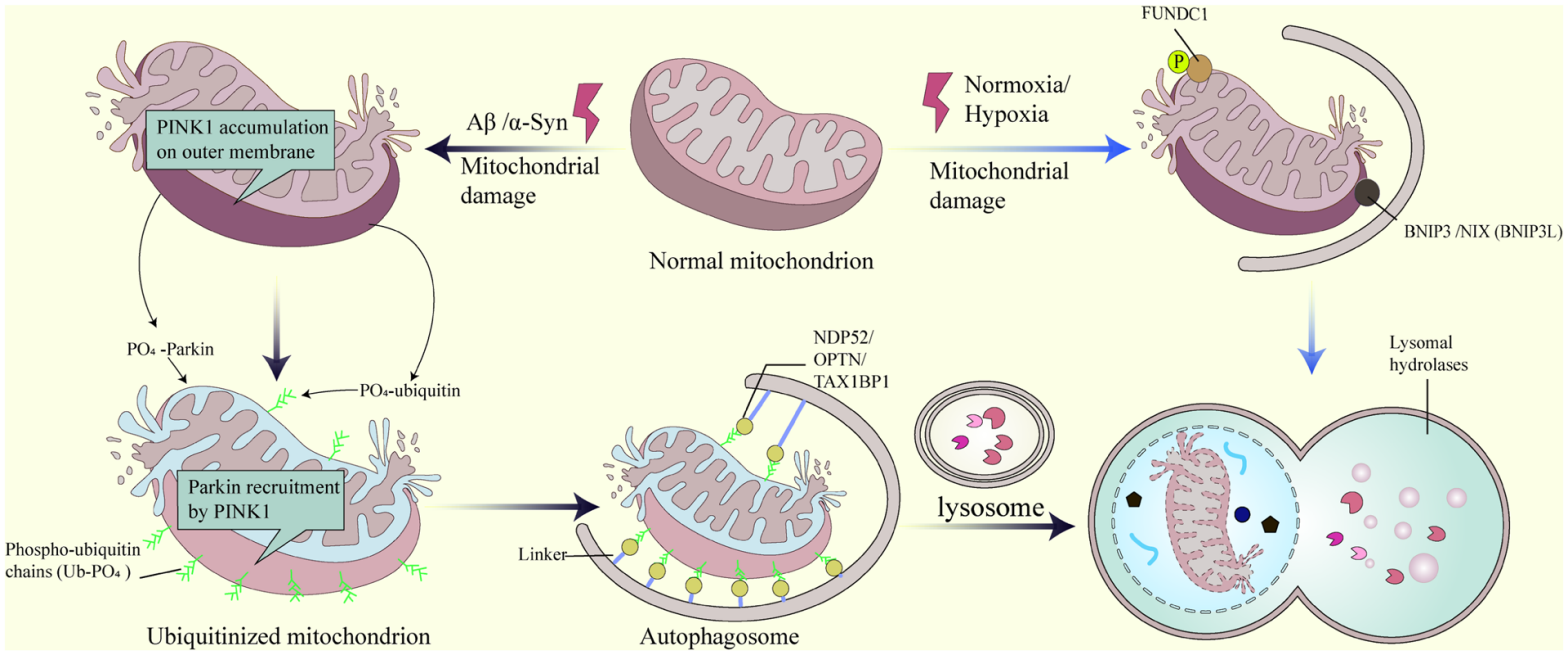
Schematic diagram of two principal pathways of mitophagy activation. Ubiquitin-dependent pathway: mitochondrial damage induces depolarization (loss of membrane potential) and the release of ROS and mtDNA. Consequently, PINK1 fails to translocate to the inner membrane and instead stabilizes and accumulates on the outer mitochondrial membrane (OMM), where it undergoes autophosphorylation and becomes activated. Activated PINK1 phosphorylates the cytosolic E3 ubiquitin ligase Parkin and recruits it to the surface of damaged mitochondria. Activated Parkin subsequently ubiquitinates multiple proteins on the OMM. These ubiquitin chains recruit autophagy adaptor proteins (e.g., p62), which simultaneously bind to LC3 on the autophagosome membrane, thereby facilitating sequestration of the damaged mitochondrion within the autophagosome for subsequent lysosomal degradation. Ubiquitin-independent pathway: this pathway involves receptors such as FUNDC1, BNIP3L, and BNIP3, which contain an LC3-interacting region (LIR). Upon receiving specific signals (e.g., hypoxia or oxidative stress), these receptors undergo conformational changes that expose the LIR domain, enabling direct binding to LC3. This interaction anchors the mitochondrion to the forming autophagosome. Timely clearance of damaged mitochondria prevents excessive ROS production and inappropriate activation of apoptosis, thereby maintaining cellular homeostasis

Mitophagy represents a critical mitochondrial quality control mechanism and has emerged as a potential therapeutic entry point in neurodegenerative disorders such as AD and PD [24, 25]. The molecular mechanisms of mitophagy mainly involve two pathways: PINK1/Parkin-dependent ubiquitination pathway and receptor-mediated, ubiquitin-independent pathway (Fig. 1) [26–29]. Dysfunction of the core mitophagy pathways represents an early pathological event in AD and PD. Preclinical evidence demonstrates that, in PD models, restoring Parkin expression through genetic editing can effectively eliminate damaged mitochondria and significantly increase the survival of dopaminergic neurons in the substantia nigra, confirming that Parkin deficiency is a key factor driving PD-associated neurodegeneration [30, 31]. Furthermore, studies in APP/PS1 transgenic mice, 3 × Tg-AD mice, and SAMP8 mice have demonstrated that improving the PINK1/Parkin-mediated mitophagy exerts protective effects against AD pathogenesis [32–34]. However, the translational feasibility of targeting this pathway remains uncertain. Gene-editing approaches, while mechanistically compelling, face substantial barriers related to delivery efficiency, long-term safety, and cell-type specificity in the human brain.

Similarly, pharmacological strategies aimed at enhancing the receptor-mediated mitophagy, including FUN14 domain-containing protein 1 (FUNDC1)-dependent pathways, have demonstrated protective effects in experimental models of AD and PD. A recent study integrating analyses of clinical samples, PD mouse models, and cellular assays revealed a significant reduction of plasma FUNDC1 level in both PD patients and model mice [35]. However, this alteration was reversed in PD patients and model mice following treatment with empagliflozin, an SGLT2 (sodium–glucose cotransporter 2) inhibitor, with concomitant restoration of mitochondrial ultrastructure and membrane potential. This reveals that the reduction of FUNDC1 level can serve as a potential biomarker with clinical relevance. Dysfunction of FUNDC1-mediated mitophagy is closely associated with key pathological hallmarks of AD, including Aβ accumulation, tau pathology, oxidative stress, and neuronal loss, as demonstrated in AD animal models and in vitro cellular systems [29]. Nevertheless, whether these effects are mitophagy-specific or secondary to systemic metabolic changes remains to be clarified. Furthermore, selective pharmacological activators of FUNDC1 are currently lacking, underscoring a major gap between mechanistic insight and drug development.

Collectively, these studies underscore the central role of mitophagy in the pathogenesis, progression, and therapeutic targeting of both AD and PD. Its therapeutic significance arises not only from the targeted modulation of mitochondrial dysfunction—an early and shared pathological event—but also from its position at the intersection of multiple pathogenic networks, including impaired proteostasis, excessive oxidative stress, neuroinflammation, and synaptic energy deficits. Therefore, elucidating the physiological role of mitophagy in AD and PD and developing mitophagy-targeted therapeutics hold substantial potential to attenuate disease progression.

### Changes in pathological proteins during the process of mitophagy

Although mitophagy does not directly degrade Aβ or α-Syn, it influences the mitochondrial stress environment conducive to protein misfolding. Mitochondrial dysfunction-induced ROS overproduction and redox imbalance facilitate aberrant APP processing and Aβ generation, suggesting that mitophagy enhancement may indirectly suppress the amyloidogenic cascades [36]. Experimental studies demonstrate that genetic or pharmacological augmentation of mitophagy, including NAD⁺ precursor supplementation, reduces Aβ burden and improves cognitive performance in preclinical models [37]. However, the translational relevance of these findings remains to be fully established. The extent to which NAD⁺ augmentation specifically enhances mitophagy in human neurons, and whether long-term activation may disrupt the metabolic homeostasis, warrant careful evaluation.

Mitophagy also intersects with α-Syn pathology in PD. Excess α-Syn impairs the mitophagic flux, while mitochondrial dysfunction promotes α-Syn aggregation, forming a pathogenic feed-forward loop. This bidirectional relationship raises the possibility that targeting mitophagy could interrupt the disease amplification cycle [38, 39]. USP30, a mitochondrial deubiquitinase that counteracts the Parkin-mediated ubiquitination, has emerged as a potential druggable target [40]. Recent studies have demonstrated that genetic ablation of USP30 enhances mitophagy, reduces the accumulation of phosphorylated Ser129 α-Syn, protects dopaminergic neurons, and ameliorates motor deficits [41]. Moreover, α-Syn overexpression per se does not alter the basal mitophagy, indicating that the observed protective effects are not attributable to nonspecific mechanisms, but rather result directly from the USP30-mediated regulation of mitophagy.

The interaction between Aβ and α-Syn further highlights the appeal of targeting shared upstream pathways. In vitro and in vivo studies have demonstrated that Aβ accelerates α-Syn aggregation via heterogeneous nucleation and can induce the formation of large α-Syn oligomers that are otherwise difficult to generate [42]. However, another study demonstrated that α-Syn monomers inhibit Aβ aggregation kinetics under specific conditions, suggesting that their interaction is dependent on the concentration and the conformational state [43]. This pathological convergence suggests that the mitophagy-directed therapies may exert multiple effects across overlapping neurodegenerative spectra. However, whether mitophagy enhancement is uniformly beneficial across disease stages remains unresolved. In advanced neurodegeneration, where mitochondrial mass and bioenergetic capacity are already compromised, excessive mitophagy activation may paradoxically exacerbate neuronal vulnerability [44]. These considerations underscore the need for stage-specific therapeutic strategies and reliable biomarkers to monitor mitophagic flux in clinical settings.

## Composition and specific activation of inflammasomes

The inflammasome is a cytosolic multiprotein complex that plays a central role in the innate immune response by detecting pathogen-associated molecular patterns and DAMPs [45, 46]. The core molecular components include pattern recognition receptors (PRRs), the adaptor protein ASC (apoptosis-associated speck-like protein containing a caspase recruitment domain), and the effector protease caspase-1. PRRs function as the upstream sensors of inflammasomes, recognizing specific pathogen-associated molecular patterns and DAMP signals and thereby initiating inflammasome assembly [47, 48]. ASC functions as a critical adaptor protein that bridges upstream PRRs and downstream caspase-1 within the inflammasome complex. ASC contains two essential protein–protein interaction domains: an N-terminal pyrin domain and a C-terminal caspase activation and recruitment domain [49].

**Fig. 2:**
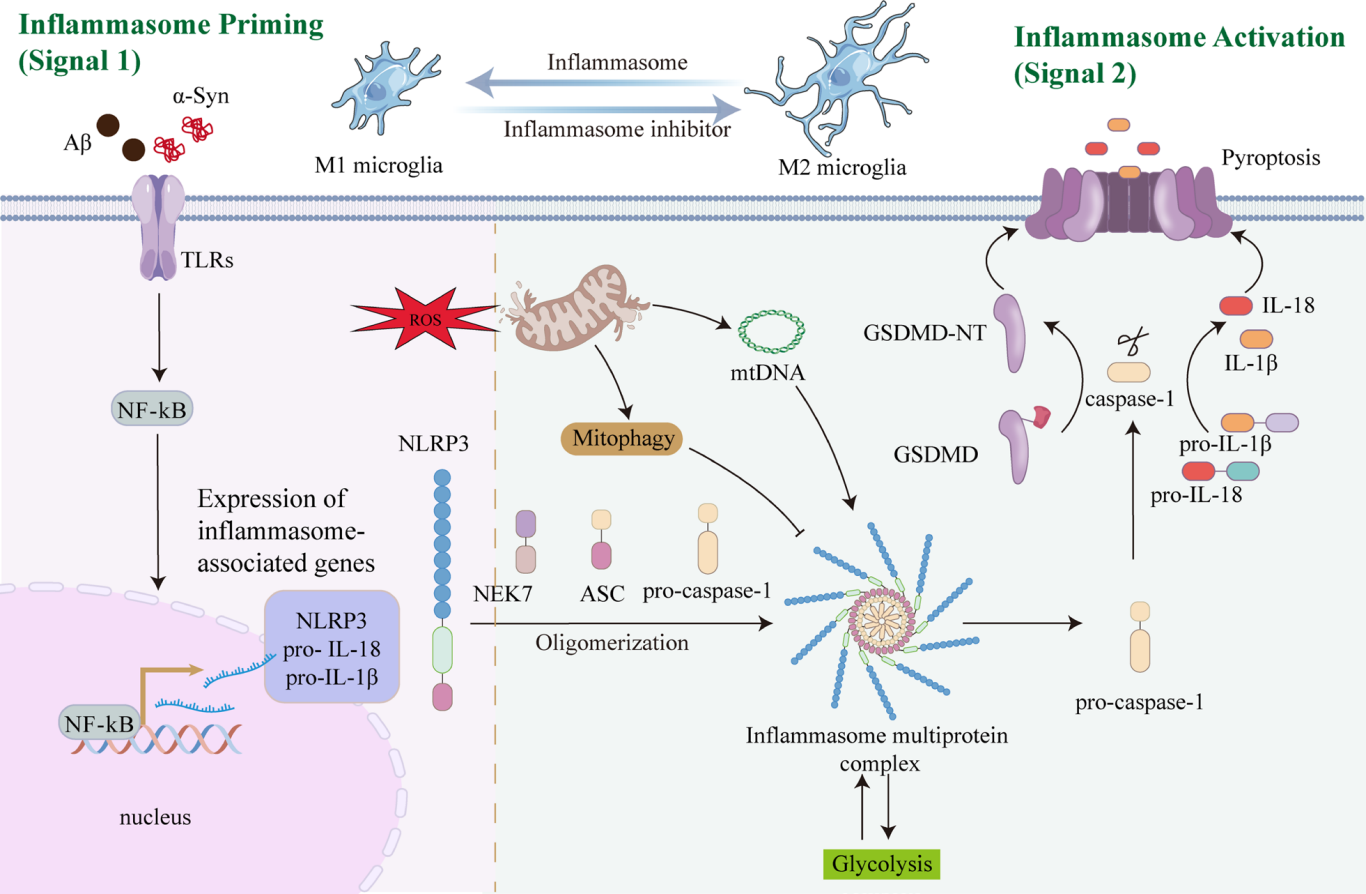
Activation of inflammasomes and their roles in neural cells (e.g., microglia). This schematic illustrates the two-step process of inflammasome activation: priming (Signal 1) and activation (Signal 2). Signal 1 (Priming): immune cells recognize α-Syn or Aβ via PRRs (e.g., TLRs), inducing nuclear translocation of NF-κB. NF-κB binds to κB sites within gene promoters, promoting transcription of NLRP3, pro-IL-1β, pro-IL-18, and pro-caspase-1. Signal 2 (Activation): diverse stimuli, including ion flux perturbations, mtDNA, and extracellular ATP, provide the second signal for NLRP3 activation. This signal induces oligomerization of NLRP3, ASC, pro-caspase-1, and NEK7 into a functional inflammasome complex. The activated caspase-1 subsequently performs two key functions: cleavage of pro-IL-1β and pro-IL-18 to generate mature cytokines, and cleavage of Gasdermin D (GSDMD). GSDMD cleavage induces pyroptosis, an inflammatory form of programmed cell death, thereby facilitating the release of mature cytokines. TLR, Toll-like receptors; NF-κB, nuclear factor-kappa B; NLRP3, NOD-like receptor pyrin domain-containing 3; mtDNA, mitochondrial DNA; ASC, apoptosis-associated speck-like protein containing a CARD; NEK7, NIMA-related kinase 7; GSDMD, gasdermin D

Inflammasomes are classified into five major types: NLRP1, NLRP3, NLRC4, AIM2, and Pyrin inflammasomes [50]. NLRP3 (NOD-, LRR-, and pyrin domain-containing protein 3) is one of the most extensively studied inflammasome-associated PRRs [51, 52]. Under physiological conditions, NLRP3 remains inactive in the cytoplasm. However, under inflammatory conditions, cytoplasmic NLRP3 expression is upregulated, potentiating innate immune responses and improving sensitivity to pathogen detection. NLRP3 activation typically requires two signals, priming (Signal 1) and activation (Signal 2) (Fig. 2) [19, 53–55].

### Specific activation of inflammasomes in AD and PD

In both AD and PD, NLRP3 inflammasome activation represents a critical mechanistic bridge between pathological protein aggregation and sustained neuroinflammation. In AD, microglial internalization of Aβ leads to lysosomal destabilization, which provides the second signal necessary for NLRP3 activation [56]. In PD, aggregated α-Syn initiates the Toll-like receptor-dependent NF-κB priming and promotes inflammasome assembly through mitochondrial dysfunction or lysosomal damage [57, 58]. These converging mechanisms position NLRP3 as a shared downstream effector of proteinopathy-driven neurotoxicity.

From a translational perspective, NLRP3 is among the most pharmacologically tractable components of the inflammasome complex. Preclinical studies have shown that pharmacological inhibition of NLRP3 attenuates IL-1β production, reduces microglial activation, and improves cognitive or motor outcomes in experimental models of AD and PD [59, 60]. Targeting upstream protein aggregates alone may not fully suppress inflammatory amplification, whereas modulation of NLRP3 offers the advantage of interrupting a common inflammatory convergence point.

However, several translational challenges remain. First, inflammasome signaling is not uniformly detrimental; early-stage microglial activation may facilitate Aβ clearance and tissue repair [61]. Therefore, complete or prolonged suppression of NLRP3 activity may impair protective immune responses. Second, systemic inhibition of NLRP3 or downstream mediators such as caspase-1 carries potential risks related to host defense and susceptibility to infection [62, 63]. Third, the lack of validated biomarkers to monitor inflammasome activation in patients complicates clinical trial design and therapeutic stratification [64].

Collectively, although inflammasome activation is pharmacologically accessible, successful clinical translation will require brain-penetrant, stage-specific modulators and biomarker-guided intervention paradigms rather than broad immunosuppression.

## Bidirectional crosstalk between mitophagy and inflammasome activation

**Fig. 3:**
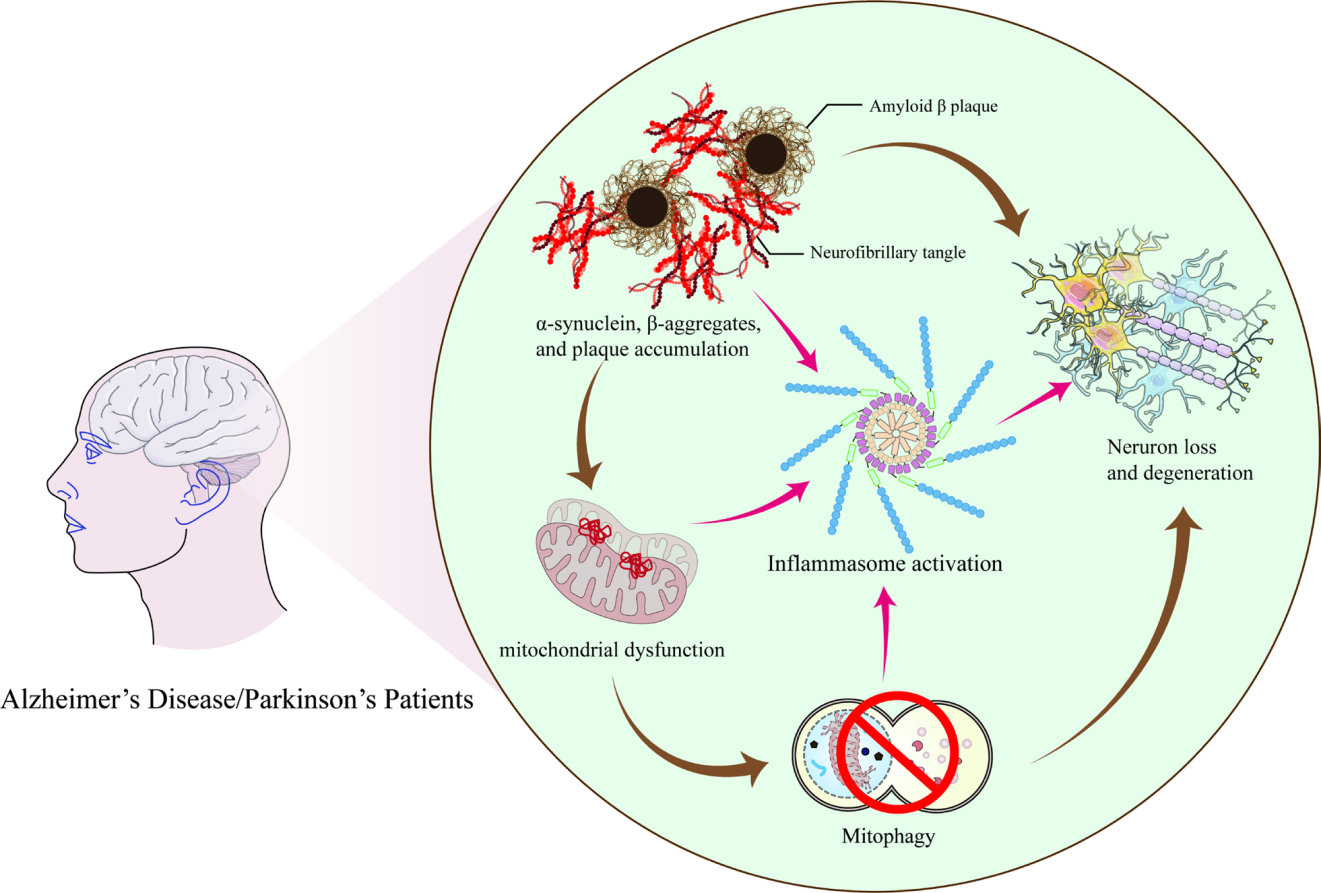
Schematic diagram of the core pathological cycle in AD and PD. This figure illustrates that abnormal protein aggregation (e.g., β-amyloid and α-synuclein) in AD and PD triggers mitochondrial dysfunction, which subsequently impairs mitophagy, activates inflammasomes, and ultimately results in neuronal loss and degeneration. The diagram highlights the shared pathogenic mechanisms underlying these two neurodegenerative disorders

Initiated by Aβ and α-Syn in AD and PD, respectively, mitophagy and NLRP3 inflammasome activation form a pathogenic feedback loop, which encompasses key pathological processes including mitochondrial damage, autophagic dysfunction, and amplification of inflammatory responses (Fig. 3). These processes exhibit synergistic interactions and mutually reinforcing effects throughout disease progression. This loop has been identified as a key pathogenic hub driving disease onset and progression [58, 65].

### Mitophagy failure promotes inflammasome activation

Aβ and α-Syn serve as primary initiators of this pathogenic regulatory loop, and their abnormal aggregation directly disrupts mitochondrial homeostasis while markedly suppressing mitophagy [16, 66]. Aβ and α-Syn specifically target and inhibit the PINK1/Parkin pathway, a central regulatory axis of mitophagy, thereby leading to significant mitophagic impairment [66]. This impairment prevents selective recognition and sequestration of damaged mitochondria, as well as their delivery to lysosomes for degradation, resulting in abnormal accumulation of dysfunctional mitochondria within cells. In parallel, Aβ induces mitochondrial fragmentation and dysfunction. Following phagocytosis by microglia, α-Syn aggregates further compromise the lysosomal membrane integrity and promote the release of cathepsin B, exacerbating mitochondrial damage through dual pathogenic effects [67, 68].

The aberrant accumulation of damaged mitochondria can trigger NLRP3 inflammasome activation through the release of DAMPs, reinforcing this pathogenic feedback loop [18, 69]. The dysfunctional mitochondria release substantial amounts of mtDNA and ROS, which act as key secondary signals for NLRP3 inflammasome activation [70–72]. Furthermore, α-Syn oligomers with distinct topological structures generated by glycosylation, differentially modulate microglial NLRP3 inflammatory responses. In addition, the soluble α-Syn–antibody complexes can further enhance NLRP3 inflammasome activation through synergistic effects of TLR2 signaling and mitochondrial damage [73, 74].

### Inflammasome activation suppresses mitophagy

The NLRP3 inflammasome activation negatively regulates mitophagy through multiple molecular mechanisms, thereby exacerbating mitophagic dysfunction [75]. In AD and PD, NLRP3 inflammasome activation further increases mtROS production [76]. The excessive mtROS oxidize key proteins in the mitophagic pathway, thereby impairing their activity and directly reducing the mitophagy efficiency [77]. Transcription factor EB (TFEB) is a master regulator of lysosomal biogenesis and expression of autophagy-related genes [78]. Pathological NLRP3 inflammasome activation directly or indirectly blocks TFEB nuclear translocation and suppresses its transcriptional activity, inhibiting TFEB-mediated lysosomal biogenesis and autophagy. In APP/PS1 AD mice, Aβ deposition activates the NLRP3 inflammasome and disrupts the autophagy–lysosome pathway, accompanied by reduced TFEB nuclear entry and downregulated lysosomal gene expression [79]. Conversely, the p38-TFEB axis inhibits chaperone-mediated, autophagy-dependent NLRP3 degradation to promote microglial inflammatory activation in PD [78]. Nuclear translocation and activation of TFEB upregulates mitophagy-related genes (e.g., *Parkin*, *LC3*), thereby promoting mitophagy [80]. Studies in PD patients and mouse models further indicate that Parkin deficiency or inactivation prevents the ubiquitin-mediated proteasomal degradation of the NLRP3 inflammasome, leading to its accumulation [81]. This defect also results in the accumulation of PARIS (Parkin-interacting substrate), a downstream substrate of Parkin, which activates the NLRP3 inflammasome via mtROS production and ultimately induces dopaminergic neuronal death through caspase-1 activation. ASC specks, aggregates formed by the inflammasome adaptor protein ASC, are upregulated in the brains of PD patients [82]. These ASC specks propagate intercellularly in a prion-like manner and persistently activate the NLRP3 inflammasome in the extracellular milieu. Persistent inflammatory signaling amplifies the aforementioned mitophagy-inhibiting mechanisms, forming a self-reinforcing pathogenic feedback loop that further drives disease progression.

Sustained activation of this pathogenic regulatory loop results in marked oxidative stress, persistent amplification of chronic neuroinflammation, and progressive neuronal damage, ultimately driving the development and progression of core pathological phenotypes, including cognitive impairment in AD and motor dysfunction in PD [83, 84]. Therefore, targeting key regulatory nodes within this pathogenic regulatory loop, such as restoring mitophagy and inhibiting NLRP3 inflammasome activation, may represent a central strategy for the development of novel therapeutics for neurodegenerative diseases, thereby providing new directions for translational research [66, 75].

### Regulatory roles of glial cells

Microglia and astrocytes constitute the cellular hub of the bidirectional regulatory network within the CNS, due to their roles in inflammatory regulation, aberrant protein clearance, maintenance of synaptic homeostasis, and neuronal survival [85–87].

In the early stages of AD, activation of the TREM2 (triggering receptor expressed on myeloid cells 2)–DAP12 (DNAX-activating protein 12) signaling pathway promotes microglial phagocytosis and clearance of Aβ, thereby delaying amyloid plaque formation and aggregation [88, 89]. In PS19 tauopathy mouse models expressing either wild-type TREM2 or R47H variant, unlike overexpressing the wild-type TREM2 that reduces soluble phosphorylated tau levels and mildly preserves neuronal integrity, overexpression of the R47H variant failed to significantly affect tau pathology, neurodegeneration, or microglial activation, exhibiting a loss-of-function phenotype [90]. In the APP/PS1 model, TREM2 enhances hippocampal neurogenesis by regulating microglial M2 polarization, thereby reducing Aβ plaques and tau hyperphosphorylation [91]. However, chronic or aberrant TREM2 activation may lead to microglial dysfunction [92]. Currently, a variety of drugs have been shown to increase the expression of TREM2, such as the natural compounds Celastrol and Ouabain [93, 94].

Complement component 1q (C1q), a central component of the complement system, is produced in large quantities by various cell types, including macrophages, dendritic cells, endothelial cells, and astrocytes, in response to inflammatory stimuli [95]. Under physiological conditions, C1q contributes to the clearance of cellular debris and apoptotic cells. However, in neurodegenerative diseases, abnormal C1q deposition is associated with dysregulated synaptic pruning [96]. A study revealed that the circulating extracellular vesicles containing C1q are delivered to neurons, thereby activating the JAK2–STAT1 signaling pathway [97]. This activation upregulates β-secretase expression, subsequently enhancing β-cleavage of amyloid precursor protein within lipid rafts. These events result in a substantial increase in Aβ production, thereby exacerbating AD pathogenesis. ANX005, a humanized anti-C1q monoclonal antibody, can effectively cross the blood–brain barrier (BBB), significantly reduce the C1q level in cerebrospinal fluid (CSF), and simultaneously improve biomarkers related to neuroinflammation in a Phase I clinical trial of healthy volunteers [98, 99]. In the pathological microenvironment of PD, activated astrocytes, in addition to microglia, secrete complement C3 [100]. C3 is a central component of the complement system. The cleavage product C3a binds to the C3a receptor (C3aR) on neurons and microglia, forming the C3aR–C3a signaling axis [101]. The complement inhibitors RaCI (a C5 inhibitor) and Cp20 (a C3 inhibitor) can rescue the α-synuclein-dependent cytotoxicity [102]. The astrocyte-derived C3 can also activate C3aR on microglia, thereby promoting inflammatory responses and neuronal apoptosis [100]. Partial deficiency of Cntnap4 (which is connected to both autism and PD) exacerbates α-Syn pathology through the astrocyte‒microglia C3‒C3aR pathway. Moreover, C3 expression is markedly elevated in astrocytes within the substantia nigra of PD patients, and this elevation correlates with the severity of neuronal loss. These findings suggest that the C3aR‒C3a axis represents a potential therapeutic target for PD.

## Pathological association between AD and PD: commonalities and distinctions

Protein misfolding represents a fundamental pathogenic feature of both AD and PD. The aggregates of misfolded protein adopt a β-sheet-rich amyloid structure that contributes to neurotoxicity by disrupting synaptic integrity, inducing oxidative stress, and promoting neurodegenerative pathways [103]. A large-scale cohort study (*n* = 2315) conducted between 2024 and 2025 provided initial in vivo evidence that misfolded α-Syn is prevalent in PD and strongly correlates with AD biomarkers [104]. In individuals with mild cognitive impairment and AD, CSF α-Syn concentrations are markedly elevated and exhibit synergistic associations with Aβ and tau accumulation. In PD, α-Syn aggregation disrupts neuronal homeostasis through toxic interactions with mitochondrial dysfunction, impairing mitochondrial respiratory chain activity and ultimately leading to energy depletion and cell death [105]. Although α-Syn aggregation is not a primary pathological hallmark of AD, it may explain why approximately 20%–30% of AD patients exhibit Lewy body pathology, suggesting shared molecular cascades of protein aggregation across neurodegenerative disorders (Fig. 3) [106, 107].

Due to the structural similarity between Aβ and α-Syn, AD and PD exhibit a pathological coupling characterized by mitophagy defects and excessive activation of the NLRP3 inflammasome. In both diseases, dysfunction of the PINK1/Parkin pathway acts as a key node of autophagic impairment, leading to mitochondrial dysfunction, which in turn activates microglial NLRP3, drives chronic neuroinflammation and neuronal pyroptosis, and accelerates disease progression. However, AD and PD also exhibit disease-specific divergence, which primarily arises from differences in the upstream pathological proteins, the affected brain regions, and the clinical phenotypes, as detailed in Table 1.

**Table 1:** Comparison of the mitophagy–inflammasome axis features between Alzheimer’s disease (AD) and Parkinson’s disease (PD)

Dimension	Shared features	AD-Specific	PD-Specific
Core protein triggers	Pathological protein aggregates induce mitochondrial damage and inhibit mitophagy	Aβ oligomers/plaques, phosphorylated tau	α-Syn oligomers, lewy bodies
Mitochondrial dysfunction	Increased ROS, reduced mitochondrial function	Energy metabolism deficits secondary to Aβ and tau pathology	Complex I inhibition [108], oxidative phosphorylation deficits, ROS
Mitophagy defects	Mainly PINK1/Parkin pathway impairment leading to accumulation of damaged mitochondria	Lysosomal acidification defects, autophagic flux blockade, APP-CTFβ accumulation [109]	PINK1/Parkin mutations or functional inactivation, strongly suppressing mitophagy flux [110]
Inflammasome activation	NLRP3-dependent cascade (caspase-1 activation, mature IL-1β/IL-18 secretion, pyroptosis)	Microglial NLRP3 dominant, ASC speck-mediated inflammatory spread [59]	Microglia and neurons co-activate NLRP3, stronger inflammation in substantia nigra [100]
Bidirectional loop	Mitophagy defects cause release of DAMPs, further activating NLRP3 and creating a vicious cycle of autophagy inhibition and inflammation	Aβ induces lysosomal damage, triggering mitochondrial stress and inflammation, which in turn promotes further Aβ accumulation [111]	α-Syn aggregation causes mitochondrial membrane damage and VDAC interaction, generating ROS and inflammation, which promotes further α-Syn accumulation [112]
Affected cells/brain regions	Neuronal loss, microglial and astrocytic activation	Hippocampal/cortical neurons, cognitive circuits	Substantia nigra dopaminergic neurons, motor control circuits

VDAC, Voltage-dependent anion channel; APP-CTFβ, β-carboxyl-terminal fragment of amyloid-β precursor protein

### AD: Aβ accumulation driven by lysosomal dysfunction

Evidence indicates that the lysosomal acidification capacity is markedly reduced in AD animal models prior to extracellular amyloid deposition [111]. This deficit correlates with a substantial reduction in the vacuolar-type ATPase (v-ATPase) activity [113]. V-ATPase is a proton pump located on lysosomal membrane, which translocates protons into the lysosomal lumen to maintain an acidic microenvironment (normal pH ~ 4.5) [114]. Studies have demonstrated that the phosphorylated amyloid precursor protein β-C-terminal fragment (APP-βCTF; phosphorylated at Tyr682) selectively interacts with the cytoplasmic domain of the V0a1 subunit of v-ATPase. This interaction competitively inhibits v-ATPase activity, leading to lysosomal alkalinization, with pH values exceeding 6.0 [115]. This mechanism has been confirmed both in Down syndrome, which is strongly associated with early-onset AD, and in AD model mice [115].

Elevated lysosomal pH significantly impairs the degradative capacity via the autophagic–lysosomal pathway [114]. Undigested APP-βCTF fragments accumulate within autophagosomes [111]. Further processing of these APP-βCTF fragments by BACE1 (β-site amyloid precursor protein-cleaving enzyme 1) and γ-secretase, increases Aβ production and reinforces a self-perpetuating pathogenic cycle [109]. Concurrently, impaired lysosomal acidification causes inactivation of multiple lysosomal hydrolases, reducing the degradation of Aβ and other protein aggregates and thereby exacerbating Aβ accumulation [116], ultimately disrupting the autophagic flux. Despite normal formation of autophagosomes and sequestration of cellular contents, lysosomal dysfunction impairs fusion of autophagosomes with lysosomes or prevents proper acidification of the resulting autolysosomes, thereby hindering cargo degradation [111]. These dysfunctional structures, termed “ineffective autolysosomes”, represent early sites of amyloid deposition [113]. Collectively, these findings indicate that lysosomal dysfunction is an early and critical driver of Aβ accumulation in AD.

### PD: α-Syn-mediated toxicity

Aberrant aggregates of α-syn, particularly oligomers, can directly target mitochondria, disrupting mitochondrial membranes and inhibiting mitophagy [112, 117]. Studies have shown that α-Syn can translocate to mitochondria and form a complex with the voltage-dependent anion channel (VDAC) on the outer mitochondrial membrane, partially blocking the VDAC pore and inducing mitochondrial calcium overload [112]. HK2p, a small membrane-binding peptide derived from the N-terminus of hexokinase 2, prevents α-Syn membrane binding, thereby reducing the α-Syn–VDAC complex formation and VDAC blockade and inhibiting α-Syn-induced mitochondrial calcium influx.

Beyond direct mitochondrial injury, pathological α-Syn potently activates microglia and drives pro-inflammatory signaling [118]. Evidence from α-Syn preformed fibril (α-Syn-PFF) mouse models and post-mortem brain samples from PD patients indicates that pathological α-Syn aggregation induces DNA damage in microglia, activating the cGAS-STING pathway and eliciting neuroinflammation [119]. In another study, both in vitro and in vivo experiments confirmed that targeted inhibition of STING by C-176 significantly suppressed the NLRP3 inflammasome activation in PD models [120]. Although many inhibitors with potent efficacy in vitro (e.g., RU.521, H-151) failed to efficiently penetrate the CNS, the potential of the STING signaling pathway as a therapeutic target in PD cannot be overlooked [121].

Notably, neurons act as an active mediator rather than a passive bystander in the NLRP3 inflammasome-associated pathogenesis. The neuronal NLRP3 can be directly activated by α-Syn oligomers [122]. This process occurs independently of microglia-derived paracrine signaling, constituting an intrinsic pathogenic circuit. The neuronal NLRP3 inflammasome is not merely a downstream effector, but rather an integrative hub and amplifier of upstream pathological events, including α-Syn aggregation, loss of Parkin function, and mitochondrial dysfunction [123]. By driving pyroptosis, exacerbating mitochondrial dysfunction, impairing autophagy, and facilitating the propagation of pathological proteins, it directly contributes to and accelerates the degeneration of dopaminergic neurons. Future studies should further delineate the precise molecular switches governing the neuronal NLRP3 activation, clarify its crosstalk with microglial NLRP3, and explore strategies to achieve cell type-specific therapeutic targeting.

## Strategic targeting of the mitophagy–inflammasome axis

**Fig. 4:**
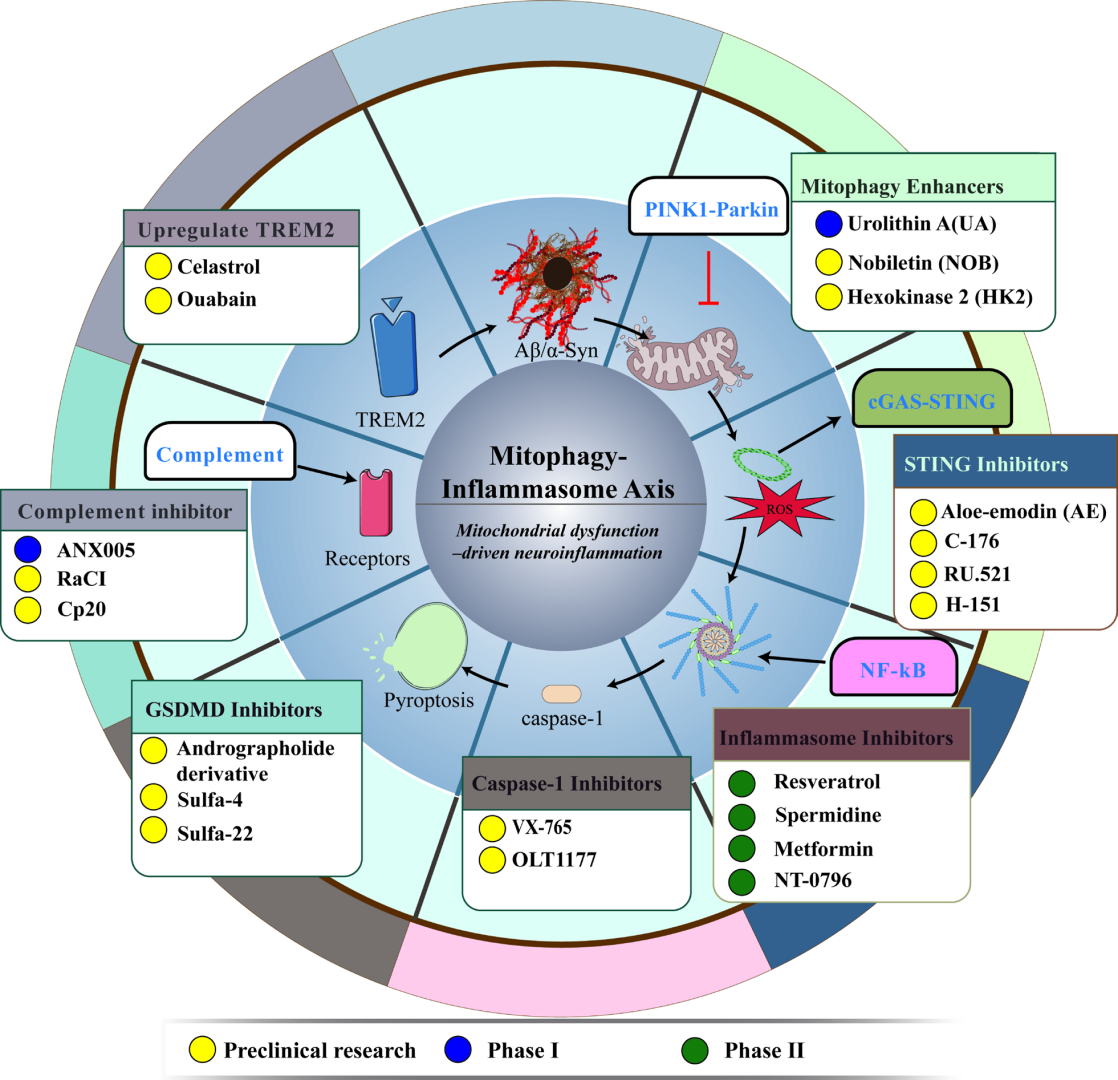
An integrative therapeutic landscape targeting mitochondrial dysfunction, inflammation, and innate immune signaling in AD/PD. Candidate therapeutic strategies targeting key molecular nodes including PINK1–parkin, mtROS/mtDNA damage, NLRP3 inflammasome, caspase-1, GSDMD, cGAS–STING, TREM2, and the complement system are shown. Different developmental stages (preclinical, phase I, and phase II) of the therapeutics are indicated in different colors

Given the complexity of neurodegenerative diseases, single-target therapies often yield suboptimal outcomes [124]. In recent years, significant advances have been made in the development of therapeutics targeting the bidirectional mitophagy–inflammasome axis [125]. Beyond alleviating mitochondrial stress and autophagy imbalance, suppressing inflammasome propagation and the downstream inflammatory signaling may represent a viable therapeutic strategy for AD. This section summarizes recent advances in the pharmacological modulation of the interplay between inflammasomes and mitophagy, emphasizing how various agents influence disease-related pathological processes (Fig. 4). A summary of these findings is presented in Table 2.

**Table 2:** Summary of agents targeting the mitophagy–inflammasome axis in AD and PD models

Agents/drugs	Underlying mechanisms	Effect	Development phase	Experimental models	References
Urolithin A (UA)	Regulates cathepsin Z to restore lysosomal function, downregulates LAMP1, upregulates Parkin/BNIP3, restores PINK1/ubiquitin expression	Reduces Aβ accumulation in frontal cortex, decreases tau phosphorylation (Thr181/Thr231), suppresses neuroinflammation (downregulation of IL-1β/NLRP3/AIM2)	Phase I [145]	APP/PS1, 3×TgAD, 3×TgAD/Polβ⁺/⁻ mice; HMC3 human microglia; HEK293 APP-Swedish cells	[126]
ALT001	Activates ULK1/Rab9-dependent alternative mitophagy	Restores Aβ phagocytic capacity, downregulates pro-inflammatory cytokines, reverses the DAM phenotype, and reduces the number of damaged mitochondria	Preclinical	Primary microglia, BV2, HMC3, human embryonic stem cell-derived microglia	[146]
Nobiletin (NOB)	Activates Bmal1/Nrf2 pathway to upregulate PINK1 and Parkin expression	Promotes mitophagy and increases expression of autophagy markers Beclin-1 and LC3-II, restoring cellular homeostasis	Preclinical	Wistar albino rats	[147]
Oleanolic acid	Activates JNK to promote Sp1 upregulation and nuclear translocation; Sp1 binds the DJ-1 promoter to enhance transcription	Promotes autophagic degradation of damaged mitochondria, alleviates mitochondrial dysfunction and ROS accumulation, protects dopaminergic neurons	Preclinical	SH-SY5Y cells, C57BL/6 mice	[148]
Hexokinase 2 (HK2)	Activates the PINK1/HK2 pathway	Removes damaged mitochondria, maintains astrocyte function, suppresses inflammatory cytokine production	Preclinical	Astrocytes	[149]
Chuanxiong–Danggui (herbal pair)	Upregulates the PGAM5/FUNDC1-mediated mitophagy	Promotes clearance of damaged mitochondria, reduces Aβ deposition, inhibits neuronal apoptosis, and restores synaptic function	Preclinical	APP/PS1 mice, HT-22 cells	[150]
Dihydro-resveratrol (DHR)	Activates the Bnip3-dependent mitophagy and ULK phosphorylation, inhibits NLRP3 inflammasome activation	Improves cognitive deficits and reduces amyloid precursor protein (APP) pathology	Preclinical	C57BL/6 mice, 5×FAD transgenic AD mice, microglia	[151]
Aloe-emodin (AE)	Activates the AMPK/PGC-1α/SIRT3 pathway	Regulates mitochondrial dynamics (Opa1, Drp1) and expression of mitophagy markers (Beclin1, LC3, P62, PINK1, Parkin)	Preclinical research	APP/PS1 and C57BL/6 J mice, HT-22 cells	[152]
Spautin-1	Binds TOMM complex, inhibits mitochondrial import and PARL-mediated cleavage of PINK1, stabilizing full-length PINK1 on the outer mitochondrial membrane	Promotes PINK1–PRKN-dependent mitophagy	Preclinical research	HeLa/SH-SY5Y cells, HsMAPT/TauP301L *C. elegans*	[153]
Andrographolide derivative (ADA)	Activates the SIRT3–FOXO3a signaling pathway	Enhances mitochondrial–lysosomal degradation, reduces DAMPs, and suppresses NLRP3 inflammasome activation	Preclinical research	ApoE3/ApoE4 mice, ApoE4-transfected BV2 and HT-22 cells	[154]
OAB-14	Increases SIRT3 expression and activity	Improves mitochondrial morphology and dynamics, enhances mitochondrial function, reduces mitochondrial Aβ accumulation	Preclinical research	APP/PS1 transgenic mice, N2a/APP cells	[155]
MCC950	Directly interacts with the Walker B motif in the NLRP3 NACHT domain	Blocks ATP hydrolysis, inhibits NLRP3 oligomerization and inflammasome assembly, reducing IL-1β release and pyroptosis	Preclinical research	Mouse bone marrow-derived macrophages, human monocyte-derived macrophages	[134]
VX-765	Inhibits the Nlrp1–Casp1–Casp6 signaling pathway	Reverses neuroinflammation and prevents progressive Aβ deposition	Preclinical research	J20 transgenic mice, human neurons	[156]
OLT1177	Blocks the Aβ-induced NLRP3 inflammasome assembly and activation, reducing caspase-1 activation	Decreases IL-1β maturation and release, restores synaptic and neuronal structure	Preclinical research	C57BL/6 J WT mice, APP/PS1ΔE9 mice	[157]
Metformin	Modulates the AMPK/mTOR/S6K/Bace1 pathway (reduces Bace1 and Aβ generation) and the AMPK/P65 NF-κB pathway (inhibits NF-κB activation)	Activates mitophagy and suppresses neuroinflammation	Phase II [158]	APP/PS1 transgenic mice	[159]
Resveratrol	Activates the SIRT1–PGC-1α axis, inhibits NF-κB and NLRP3 activation	Reduces neuroinflammation and Aβ toxicity, upregulates autophagy-related proteins	Phase I/II [160]	3×Tg-AD mice	[143]
Spermidine	Regulates Beclin1 and ATG16L1 expression, inhibits NF-κB signaling, reduces ASC oligomerization	Activates autophagy, suppresses inflammasome assembly, downregulates pro-inflammatory genes, reduces soluble Aβ	Phase I/II [161]	APP/PS1 mouse model	[162]

### Mitophagy enhancers

The therapeutic potential of mitophagy enhancers has been systematically validated across multiple experimental models. Urolithin A (UA) significantly improves cognitive function in AD mouse models and alleviates motor dysfunction and dopaminergic neurodegeneration in PD models by activating mitophagy and restoring lysosomal function [126, 127]. The natural compound galangin reverses the Aβ-induced mitophagy inhibition and rescues organoid growth defects in human brain organoid models [128]. Collectively, these findings suggest that enhancing mitophagy is not merely an act of “accelerating waste clearance”, but rather a process that restructures the intrinsic stress resistance and metabolic resilience of neurons. Recent studies have demonstrated that moderate activation of mitophagy can concomitantly enhance mitochondrial biogenesis (via PGC-1α), antioxidant defense (via Nrf2), and lysosomal function (via TFEB), thereby forming a synergistic protective network with efficacy surpassing that of single-target therapies [129, 130]. However, translating this therapeutic potential into clinical practice remains challenged by three fundamental obstacles as follows.

*The spatiotemporal precision challenge.* Mitophagy exhibits a strict dose–response relationship; excessive or temporally inappropriate activation may lead to unintended clearance of healthy mitochondria, inducing an energy crisis and even apoptosis. Therefore, therapeutic strategies with cell-type specificity, precise subcellular localization, and dynamic responsiveness are required [131]. Emerging technologies, such as autophagy-tethering compounds, aim to engineer bifunctional small molecules in which one moiety specifically binds mitochondrial membrane proteins (e.g., TOM20) and the other engages LC3, thereby bypassing the PINK1/Parkin pathway to achieve targeted “physical tethering”. These compounds have demonstrated superior specificity and safety compared with conventional mitophagy inducers in PD and Down syndrome models [132].

*Lack of biomarkers.* Reliable and non-invasive approaches for dynamic monitoring of mitochondrial autophagic flux in vivo remain unavailable [44]. Although p62, LC3-II, and mtDNA fragments in CSF have been proposed as potential mitophagy-related biomarkers, their sensitivity and specificity require validation in large-scale cohorts [133]. For example, one study quantified mitophagy- and lysosome-related biomarkers (PINK1, BNIP3L [BCL2 interacting protein 3], and TFEB) in the CSF and serum of 246 individuals and found that patients with AD exhibited significant alterations of these markers [133]. Mitophagic flux represents a critical parameter for the overall degradative efficiency of mitophagy, as static measurements of autophagosome abundance cannot distinguish between enhanced autophagic induction and impaired degradation. Accurate assessment of autophagic activity therefore requires dynamic monitoring of the entire process, from autophagosome formation to lysosomal degradation. The absence of validated biomarkers in clinical trials severely hampers patient stratification, efficacy assessment, and decision-making throughout the drug development process.

*BBB delivery bottleneck.* The brain is tightly protected by the BBB. Although this barrier effectively prevents the entry of harmful substances, it also restricts the penetration of most therapeutic agents into the CNS, resulting in low cerebral bioavailability and short half-lives for many mitophagy-targeting candidates (e.g., rapamycin derivatives and NAD^+^ precursors), thereby limiting their ability to achieve therapeutically effective concentrations [44].

### Inflammasome inhibitors

Inflammasome inhibitors can effectively suppress the inflammatory cascade and pyroptosis, thereby mitigating neuronal damage and slowing disease progression. MCC950 (also known as CP-456773) is a potent and specific inhibitor of the NLRP3 inflammasome [134]. Studies in AD and PD models have validated that MCC950 reduces neuroinflammation and neurodegeneration by inhibiting the NLRP3 inflammasome [135, 136]. Another natural compound, asiaticoside, exerts neuroprotective effects by directly inhibiting NLRP3 inflammasome assembly [14]. In the field of synthetic therapeutics, highly selective and BBB-penetrant candidate molecules have advanced into clinical development. NT-0796, a brain-penetrant NLRP3 inhibitor that demonstrated robust anti-neuroinflammatory activity in preclinical models, has been designated a clinical candidate for the treatment of neuroinflammatory disorders such as AD and PD [137]. In addition, candidate inhibitors of downstream Gasdermin D cleavage (e.g., Sulfa-4 and Sulfa-22) have also exhibited potent anti-inflammatory effects in the APP/PS1 mouse model [138].

At the clinical translation stage, multiple Phase I/II trials are evaluating the safety, pharmacokinetics, and biomarker effects of NLRP3 inhibitors in patients with early-stage AD. For example, DFV890 is currently undergoing clinical evaluation of its effects on CSF levels of IL-1β and IL-18, as well as on neuroimaging biomarkers [139].

However, significant challenges remain. First, the BBB represents a major obstacle. Second, chronic inhibition of innate immunity may increase the risk of infection, necessitating precise optimization of dosage and treatment duration [140]. Third, patient stratification is critical, requiring accurate screening based on biomarkers such as IL-1β, ASC specks, and specific gene expression profiles [141].

In summary, NLRP3 inflammasome inhibitors represent a transformative therapeutic strategy supported by strong mechanistic rationale and robust preclinical evidence. Translation of these inhibitors may not only reshape the therapeutic landscape of AD and PD, but also establish a broader paradigm for understanding and intervening in aging-related chronic neuroinflammation.

### Dual-modality approaches

Within the pathological framework of AD and PD, single-target intervention strategies—particularly traditional anti-inflammatory drugs or antioxidants that merely ameliorate mitochondrial function—face fundamental limitations [76]. Therefore, combining mitochondrial agonists with inflammasome inhibitors to target the “mitochondria–inflammation” axis, has the potential to achieve synergistic therapeutic effects. Preclinical studies have provided robust evidence supporting this combinatorial strategy.

In 5 × FAD transgenic AD mice, co-administration of MitoQ and Ciliatoside A (a natural NLRP3 inhibitor) reduced hippocampal Aβ plaque load by 62% and p-tau Ser396 level by 58%, increased the M2-to-M1 microglial polarization ratio by 2.3 folds, and restored spatial memory function reflected by shortened escape latency in the Morris water maze [142]. Furthermore, several natural products (such as resveratrol) exert dual effects by promoting mitophagy while concurrently inhibiting neuroinflammation [143, 144]. From the translational perspective, challenges persist, primarily including safety evaluation, optimization of treatment timing and dosage, and effective BBB penetration.

## Future perspectives

Given the central mechanistic role of the mitophagy–NLRP3 inflammasome axis in AD and PD, together with the preclinical evidence and druggability, NLRP3 inflammasome modulators currently hold great potential for advancing into clinical trials. Several related agents have already entered clinical development. For instance, the candidate drug NT-0796 has completed Phase I clinical trials and is initiating a Phase Ib/IIa biomarker study in PD [137]. In contrast, mitophagy enhancers and cGAS-STING pathway modulators remain in progressive developmental stages [163, 164].

A critical scientific basis propelling NLRP3 inflammasome modulators into clinical trials is the capacity to monitor target pharmacodynamic activity using CSF biomarker signatures [165], such as IL-18 and IL-1β, as well as visualizing and quantifying intracerebral neuroinflammation via positron emission tomography (PET) imaging, thereby enabling precise monitoring of the therapeutic efficacy [166]. In addition, it is crucial to accurately identify patient subgroups that may respond more significantly to NLRP3 inflammasome modulators. Screening methods based on CSF biomarkers can initially select patients with more active inflammatory responses, providing a precise basis for clinical trial enrollment [165]. In addition, comprehensive screening by integrating neuroimaging and fluid biomarkers can accurately identify patients with NLRP3 inflammasome-mediated neuroinflammation as the main pathological feature, thereby significantly improving the efficiency and the success rate of clinical trials for NLRP3 inhibitors. Future research on AD and PD should prioritize multidimensional strategies, including multi-omics and single-cell analyses, BBB-targeted delivery technologies, and multi-target synergistic therapeutic approaches, complemented by artificial intelligence (AI) to accelerate drug development [167–170].

### Multi-omics and single-cell analysis

Integrating multi-omics technologies (including genomics, epigenomics, transcriptomics, proteomics, and metabolomics) with single-cell analyses enables systematic characterization of cell-type-specific molecular landscapes in AD and PD [167, 171]. For instance, single-cell cortical transcriptomic studies have revealed both shared and distinct alterations in intercellular communication in AD and PD, facilitating identification of common susceptibility factors and underlying molecular mechanisms [171].

These approaches are expected to identify novel early-stage biomarkers, such as phosphorylated tau217 in CSF, glial fibrillary acidic protein and neurofilament light chain in plasma, as well as microglial subtype signatures defined by single-cell transcriptomics, thereby providing a basis for precise stratification during prodromal stages and optimal intervention timing [9, 172, 173]. *APOE4* homozygosity has been established as a major genetic determinant of AD, necessitating tailored research strategies and therapeutic approaches [174]. Furthermore, integrative investigations of the gut–brain–microbiome axis, combined with metagenomics, metabolomics, and AI tools, facilitate identification of next-generation biomarkers [175].

### Optimization of drug design and BBB-targeted delivery

Rational drug design enhances target selectivity through chemical structural modification. Integration with state-of-the-art technologies (e.g., nanodelivery systems) further improves CNS targeting of therapeutics [176]. Nanotechnology has demonstrated substantial potential in AD therapy, as delivering drug combinations via nanocarriers enables more efficient BBB penetration. Receptor-mediated cell-penetrating peptides (CPPs) are widely functionalized on the surface of nanocarriers. CPPs are a group of short amino acid sequences that boost the transmembrane transport of macromolecules. For the treatment of neurological diseases, lipid nanoparticles and polymeric nanoparticles modified with such peptides can traverse the BBB through transferrin receptor- or low-density lipoprotein receptor-related protein 1 (LRP1)-mediated transcytosis [177, 178]. Substantial progress has been achieved in the development of ultra-small lipoprotein carriers and exosome engineering platforms, which significantly enhance parenchymal exposure to inhibitors of Aβ, α-Syn, or LRRK2 [176].

Furthermore, gene therapy delivering therapeutic genes via viral or non-viral vectors, provides highly specific and targeted strategies for CNS disorders and has been evaluated in preclinical and clinical studies for conditions including AD, PD, spinal cord injury, and ischemic stroke [179]. Optimization of therapeutic chemical structures is also crucial for enhancing target selectivity. The multi-target drug design strategy aims to simultaneously modulate multiple pathological pathways in AD and PD to achieve superior therapeutic efficacy [168]. This approach enables synergistic intervention in disease progression by incorporating multiple bioactive moieties into a single molecule or through rational drug combinations.

### Multi-target synergistic strategies and AI-accelerated drug development

AI tools can be used to identify novel drug targets and candidate compounds by analyzing large-scale genomic, proteomic, and metabolomic datasets [169]. For instance, AI-driven multi-objective optimization models (e.g., reinforcement learning combined with graph neural networks) have demonstrated strong predictive performance for preclinical combination therapies [167, 169]. These technologies enable more efficient screening and optimization of multi-target drugs, thereby accelerating clinical trial design and facilitating the development of more effective therapeutic regimens. Epigenetic research has also gained increasing attention and is recognized as a promising therapeutic avenue for AD, with AI-powered analytical approaches providing robust support for such investigations [180, 181].

In summary, future research on AD and PD will require a systematic approach characterized by interdisciplinary integration and technological convergence. A deeper understanding of disease heterogeneity, strategies to overcome BBB limitations, multi-target synergistic interventions, and AI-enabled technological support is expected to generate transformative diagnostic and therapeutic strategies for these devastating neurodegenerative disorders.

## Conclusions

The mitophagy–inflammasome axis represents a central shared mechanism underlying neurodegeneration in AD and PD. Impaired mitophagy leads to the accumulation of damaged mitochondria, resulting in the release of DAMPs such as mtDNA and ROS. Aberrant activation of inflammasomes, particularly the NLRP3 inflammasome, further exacerbates mitochondrial dysfunction by disrupting mitochondrial quality control mechanisms. This cycle drives persistent chronic neuroinflammation and progressive neuronal loss.

Based on this shared mechanism, therapeutic strategies targeting this bidirectional regulatory network have been investigated: mitophagy agonists mitigate pathological initiation by clearing damaged mitochondria; inflammasome inhibitors abrogate inflammatory cascade signaling; and dual-target regulators mediate the synergistic “mitophagy repair–inflammation inhibition”, offering diverse therapeutic avenues for both AD and PD. With integrated application of cutting-edge technologies in disease stratification, drug development, and delivery systems, precision interventions targeting this core pathway are expected to overcome the limitations of traditional therapies, open new avenues for disease-modifying treatments in AD and PD, and ultimately enable a shift from symptomatic management to etiological intervention.

## Data Availability

Not applicable.

## Abbreviations

IL: Interleukin
NLRP3: NOD-, LRR-, and pyrin domain-containing protein 3
AD: Alzheimer’s disease
PD: Parkinson’s disease
CNS: Central nervous system
Aβ: Amyloid-β
α-Syn: α-Synuclein
DAMPs: Damage-associated molecular patterns
ROS: Reactive oxygen species
PINK1: PTEN-induced kinase 1
FUNDC1: FUN14 domain-containing protein 1
PRRs: Pattern recognition receptors
ASC: Apoptosis-associated speck-like protein
GSDMD: Gasdermin D
C1q: Complement component 1q
V-ATPase: Vacuolar-type ATPase
DAP12: DNAX-activating protein 12
APP-βCTF: Amyloid precursor protein β-C-terminal fragment
TFEB: Transcription factor EB
mtDNA: Mitochondrial DNA
